# Association of Biochemical Parameters With Hormonal Contraceptives Among Women Attending the Dschang Regional Annex Hospital, Cameroon: A Cross-Sectional Study

**DOI:** 10.7759/cureus.108799

**Published:** 2026-05-13

**Authors:** Elvire Damaris Kwapnang Nzounkeu, Jaures Arnaud Kenfack Noumedem, Jean-De-Dieu Tamokou

**Affiliations:** 1 Department of Biochemistry, University of Dschang, Dschang, CMR; 2 Department of Physiological Sciences and Biochemistry, University of Dschang, Dschang, CMR

**Keywords:** cardiovascular risks, dyslipidemia, electrolyte disturbances, hormonal contraceptives, inflammatory markers, metabolic abnormalities

## Abstract

Background

Hormonal contraceptives (HCs), including implants, oral pills, and injectables, contain synthetic estrogen and/or progestin and are widely used for pregnancy prevention and spacing. While effective, they have been linked to systemic side effects affecting metabolism, immunity, and biochemical balance, including weight gain, cardiovascular issues, and thromboembolic events. However, their effects on biochemical markers, such as lipid profiles and C-reactive protein (CRP), in our setting remain unclear. This study, therefore, aimed to assess the association between HC use and primary biochemical outcomes, including lipid profile parameters (total cholesterol, triglycerides, low-density lipoprotein cholesterol, and high-density lipoprotein cholesterol) and CRP, as well as secondary outcomes comprising serum and urinary electrolyte levels among women attending the Dschang Regional Annex Hospital, Cameroon.

Methodology

A cross-sectional study was conducted from June 2024 to June 2025 among 510 women. Data on HC use and sociodemographic characteristics were collected using structured questionnaires. Blood and urine samples were collected in sterile dry tubes and containers, respectively. Biochemical analyses were performed according to the manufacturer’s instructions to evaluate lipid profiles, inflammatory markers, and electrolyte levels.

Results

Compared with non-users, HC users exhibited significantly (p < 0.05) higher levels of total cholesterol (256.67 ± 8.57 vs. 141.12 ± 6.90 mg/dL), triglycerides (124.81 ± 4.51 vs. 84.82 ± 4.33 mg/dL), and LDL-cholesterol (158.88 ± 7.75 vs. 93.85 ± 7.36 mg/dL). CRP levels were also significantly higher among HC users (4.98 ± 0.79 vs. 3.27 ± 0.18 mg/L). Electrolyte disturbances were observed, with HC users presenting higher serum calcium (11.64 ± 0.47 vs. 9.78 ± 0.126 mg/dL), chloride (153.50 ± 7.39 vs. 103.57 ± 2.15 mmol/L), and potassium levels (6.58 ± 0.50 vs. 4.24 ± 0.09 mmol/L). Logistic regression analysis identified implant use as a significant risk factor for biochemical alterations, particularly elevated cholesterol (odds ratio (OR) = 4.57; 95% confidence interval (CI): 1.59 - 13.13; p = 0.001), LDL-cholesterol (OR = 2.80; 95% CI: 1.27 - 7.60; p = 0.001), and increased CRP levels (OR = 3.18; 95% CI: 2.07 - 10.45; p = 0.001).

Conclusions

HC use is associated with significant alterations in lipid profiles, inflammatory markers, and electrolyte balance. These findings suggest that HC may influence metabolic and inflammatory processes in users, highlighting the importance of routine biochemical monitoring among women using HC methods.

## Introduction

Hormonal contraceptives (HCs) are modern methods used to limit, delay, or space pregnancies [1]. They include implants, oral pills, and injectables containing synthetic estrogen and/or progestin, which prevent ovulation or hinder sperm penetration by thickening cervical mucus. Widely used across the world, HCs play a crucial role in improving women’s reproductive autonomy and reducing unintended pregnancies [1]. In Cameroon, the Demographic and Health Survey reports an increase in modern contraceptive use among married women from 16% in 2011 to about 19% in 2018, with hormonal methods representing a substantial share. Globally, HC use has also risen markedly, reflecting broader adoption of modern family planning strategies [2].

Despite their proven effectiveness, HCs are associated with systemic side effects, particularly affecting metabolic, immune, and biochemical functions. Reported complications include weight gain, cardiovascular disorders [3], and an increased risk of thromboembolic events [4], especially with higher estrogen doses [4]. HCs may also alter lipid metabolism through estrogen- and progestin-mediated effects on hepatic lipoprotein synthesis and clearance, potentially leading to changes in total cholesterol, triglycerides, low-density lipoprotein cholesterol (LDL-C), and high-density lipoprotein cholesterol (HDL-C) levels [5]. These lipid profile parameters are clinically important because dyslipidemia is a recognized risk factor for atherosclerosis and cardiovascular disease. In addition, HC use has been associated with elevated levels of C-reactive protein (CRP), an inflammatory biomarker linked to endothelial dysfunction, cardiovascular risk, and chronic low-grade inflammation [5]. Monitoring these biomarkers is therefore relevant for assessing the cardiometabolic safety of HC use.

In developing settings, including Cameroon, research on HC-related effects beyond reproductive health remains limited. In addition to lipid and inflammatory alterations, hormonal steroids may influence fluid and electrolyte homeostasis through activation of the renin-angiotensin-aldosterone system. Estrogens can increase hepatic angiotensinogen production, while certain progestins interact with mineralocorticoid receptors and aldosterone activity, potentially affecting sodium, potassium, chloride, and bicarbonate balance [6]. Such electrolyte disturbances may contribute to blood pressure changes, fluid retention, and cardiovascular stress. Furthermore, prolonged alterations in serum and urinary electrolytes may reflect underlying renal or endocrine adaptations associated with HC use. Despite these potential clinical implications, evidence regarding the effects of HCs on electrolyte balance remains inconsistent and insufficiently explored in sub-Saharan African populations. The scarcity of local data limits the availability of evidence-based guidance for healthcare providers and users. Therefore, this study aimed to assess the association between HC use and primary biochemical outcomes, including lipid profile parameters (total cholesterol, triglycerides, LDL-C, and HDL-C) and CRP, as well as secondary outcomes comprising serum and urinary electrolyte levels among women attending the Dschang Regional Annex Hospital. It was hypothesized that HC users would exhibit higher levels of total cholesterol, triglycerides, LDL-C, and CRP, as well as lower HDL-C levels, compared with non-users. In addition, HC use was hypothesized to be associated with alterations in serum and urinary electrolyte levels, reflecting possible hormonal effects on fluid and electrolyte regulation.

## Materials and methods

Design, study population, sampling method

A cross-sectional study was conducted from June 2024 to June 2025 at the Dschang Regional Annex Hospital and at the Research Unit of Microbiology and Antimicrobial Substances (Department of Biochemistry, University of Dschang, West Cameroon). Participants were recruited using a consecutive non-probability sampling technique, in which all eligible women attending the study sites during the data collection period were invited until the required sample size was achieved. The minimum sample size was determined a priori using the Cochran formula for cross-sectional studies, based on an assumed prevalence of HC use from previous national estimates (19%) [2], a 95% confidence level, and a 5% margin of error. The calculated minimum sample size (237 participants) was adjusted upward to account for potential non-response and incomplete data, resulting in a final sample of 510 participants. The study protocol was approved by the Regional Ethics Committee for Research and Human Health (CRERSH-OU), Bafoussam, Cameroon (ref: 400/27/03/2024/CE/CRERSH-OU/VP). Eligible participants provided written informed consent, with assent for those under 21 years. Individuals were excluded if they had liver, kidney, or cardiovascular disease; smoked; had pre-existing metabolic or inflammatory conditions; used alcohol; were pregnant; had hepatitis B, C, or D; had HIV; had malaria; had used hepatotoxic drugs recently; or had medications affecting biochemical parameters. Healthy controls underwent clinical screening, and those with diabetes, dyslipidemia, obesity (BMI ≥ 30 kg/m²), renal disease, or other metabolic disorders were excluded. Fasting glucose and lipid profiles were measured to confirm metabolic health. Participants included healthy women of reproductive age (15-49 years) attending the hospital or residing in the area who voluntarily agreed to participate. Based on the inclusion criteria (women aged 15-49 years, with HC users having a minimum of three months of continuous use) and exclusion criteria (pregnant women or those outside the specified age range), 510 participants provided written informed consent. They were divided into two groups: a healthy control group of 240 non-users and a study group of 270 hormonal contraception users.

Sample collection and biochemical analyses

Venous blood (10 mL) was collected by venipuncture from consenting women aged 15-49 years, using dry tubes and centrifuged at 3000 rpm for 10 minutes to obtain serum. Urine samples were collected in sterile containers and similarly centrifuged. Serum and urine were analyzed for CRP, lipid profile, and electrolytes using commercially available in vitro diagnostic kits based on standard enzymatic, immunoturbidimetric, and colorimetric methods, in accordance with manufacturers’ instructions and established clinical chemistry guidelines [7-9]. Analyses were performed using an automated clinical chemistry analyzer DIALAB DTN-405 (DIALAB GmbH, Wiener Neudorf, Austria), with CRP measured by immunoturbidimetric assay, lipid parameters (total cholesterol, triglycerides, HDL-C, and LDL-C) determined using enzymatic colorimetric methods, and electrolytes assessed using ion-selective electrode (ISE) techniques or validated colorimetric assays, depending on the analyte. Results were expressed in standard SI units (mg/dL for lipid, CRP, and calcium; mmol/L for other electrolyte parameters). All assays were conducted following strict laboratory quality assurance procedures, including daily calibration using manufacturer-provided calibrators, routine maintenance of the analyzer, and verification with internal quality control materials at normal and pathological levels. External quality assessment procedures were applied where available to ensure analytical accuracy and reproducibility.

Statistical analysis

Participants were classified into two groups: HC users and non-users. Data collected from questionnaires and laboratory analyses were entered, coded, and managed using Microsoft Excel (Microsoft Corporation, Redmond, Washington) and then analyzed with IBM SPSS Statistics for Windows, Version 22 (Released 2013; IBM Corp., Armonk, New York) and EpiInfo 7 (Centers for Disease Control and Prevention, Atlanta, GA, USA) software. Mean values of biochemical parameters between the two groups were compared using an independent samples t-test. Prior to analysis, the assumptions of normality and homogeneity of variances were assessed. When the assumption of equal variances was violated, Welch’s t-test was applied as an alternative. Biochemical parameters were categorized as normal or abnormal based on cut-off values provided by the assay kits. The Pearson chi-square test was applied to compare the prevalence of abnormalities between groups. The assumptions for the chi-square test were verified prior to analysis, including the requirement that at least 80% of expected cell frequencies were ≥ 5 and that no expected cell frequency was < 1. When these assumptions were not met, Fisher’s exact test was used as an alternative. Multivariate logistic regression was used to assess the association between HC use and biochemical alterations. Crude odds ratios (ORs) with 95% confidence intervals (95% CIs) were calculated using univariate logistic regression, whereas adjusted odds ratios (aORs) and p-values were obtained from multivariate logistic regression models controlling for age, physical activity, and body mass index (BMI). These factors are well established to influence lipid profiles and CRP levels. Predictor significance was assessed using the Wald chi-square test. Statistical significance was set at p < 0.05.

## Results

Sociodemographic and clinical characteristics of participants as a function of HC use

There is no significant association with age (p = 0.057), although women aged 30-40 years display slightly higher use (Table 1). In contrast, BMI is significantly associated with HC use (p < 0.001), with very high use among obese participants (96%). Parity reveals a strong relationship (p < 0.001), as contraceptive use increases with the number of children. Prior contraceptive history is also significant (p < 0.001), with the highest use among women who previously used implants. Marital status is strongly associated (p < 0.001): married women are more likely to use contraceptives than single women. Education level (p = 0.005) and profession (p < 0.001) are also significantly associated with use, with variation across groups. Overall, contraceptive use is mainly influenced by reproductive factors and socioeconomic characteristics, but not by age.

**Table 1:** Baseline sociodemographic and clinical characteristics of participants as a function of hormonal contraceptive use * Fisher’s exact test used due to small expected cell counts (< 5). The Pearson’s chi-square test was used to assess differences in the frequency of biochemical abnormalities across the two groups, with a p-value below 0.05 considered statistically significant. n: number of subjects per group.

Features		Hormonal contraceptive users n (%)	Non-users of hormonal contraceptives n (%)	Chi-square	p-value
Age groups	10–20 years n = 54	28 (51.85)	26 (48.14)	7.52	0.057
	20–30 years n = 232	119 (51.29)	113 (48.70)		
	30–40 years n = 145	89 (61.37)	56 (38.62)		
	40–50 years n = 79	34 (43.03)	45 (56.96)		
Body mass index (BMI)	Normal n = 220	110 (50.00)	110 (50.00)	18.64	< 0.001
	Overweight n = 265	136 (51.32)	129 (48.67)		
	Obesity n = 25	24 (96.00)	1 (4.00)		
Duration of contraceptive use	3–12 months	165 (100)	–	–	–
	1–2 years	44 (100)	–		
	2–4 years	36 (100)	–		
	> 4 years	25 (100)	–		
Parity	0 child n = 126	37 (29.36)	89 (70.63)	37.21	< 0.001
	1–2 children n = 153	81 (52.94)	72 (47.05)		
	3–4 children n = 133	81 (60.90)	52 (39.09)		
	≥ 5 children n = 98	71 (72.44)	27 (27.55)		
Prior contraceptive history	Implant n = 96	74 (77.08)	22 (22.92)	12.84	0.002
	Pill n = 42	22 (52.38)	20 (47.62)		
	Injection n = 17	7 (41.18)	10 (58.82)		
Marital status	Single n = 185	68 (36.75)	117 (63.24)	–	< 0.001*
	Married n = 325	202 (62.15)	123 (37.85)		
	Divorced n = 1	1 (100)	0 (0)		
Level of education	Illiterate n = 1	1 (100)	0(0)	–	< 0.001*
	Primary n = 31	20 (64.51)	11 (35.48)		
	Secondary n = 270	158 (58.51)	112 (41.48)		
	Greater n = 208	91 (43.75)	117 (56.25)		
Profession	Pupil n = 39	20 (51.28)	19 (48.71)	26.41	< 0.001
	Student n = 155	56 (36.12)	99 (63.87)		
	Formal sector n = 84	47 (55.95)	37 (44.04)		
	Informal sector n = 121	79 (65.28)	42 (34.71)		
	Housekeeping n = 111	68 (61.26)	43 (38.73)		

Distribution of mean biochemical parameters by contraceptive status

Mean biochemical values revealed significant differences between contraceptive users and non-users (Table 2). Users exhibited markedly higher levels of CRP, total cholesterol, LDL-C, triglycerides, calcium, chloride, and potassium compared with non-users (all p < 0.001). Other parameters, including HDL-C and serum sodium, showed no statistically significant differences.

**Table 2:** Distribution of mean biochemical parameters by contraceptive status Continuous variables are presented as mean ± standard error of the mean; on the same line, values marked with different superscript letters (a, b) are significantly different at p < 0.05, as determined by Student's t-test. n: number of subjects per group; HDL: high-density lipoprotein; LDL: low-density lipoprotein

Parameters	Reference values	Users n = 270	Non-users n = 240
C-reactive protein	< 6 mg/dL	4.98 ± 0.79^a^	3.27 ± 0.18^b^
Total cholesterol	< 200 mg/dL	256.67 ± 8.57^a^	141.12 ± 6.90^b^
HDL cholesterol	45–65 mg/dL	68.70 ± 6.14^a^	82.87 ± 7.01^a^
LDL cholesterol	< 150 mg/dL	158.88 ± 7.75^a^	93.85 ± 7.36^b^
Triglycerides	35–135 mg/dL	124.81 ± 4.51^a^	84.82 ± 4.33^b^
Serum calcium	8.50–10.8 mg/dL	11.64 ± 0.47^a^	9.78 ± 0.126^b^
Serum chloride	98–110 mmol/L	153.50 ± 7.39^a^	103.57 ± 2.15^b^
Serum potassium	3.60–5.50 mmol/L	6.58 ± 0.50^a ^	4.24 ± 0.09^b^
Serum sodium	136–146 mmol/L	134.61 ± 6.69^a^	133.94 ± 1.40^a^
Urine calcium	6.69–20.04 mg/dL	18.69 ± 1.93^a^	8.73 ± 0.20^b^
Urine chloride	170–250 mmol/L	188.58 ± 5.76^a^	171.70 ± 5.72^b^
Urine potassium	25–125 mmol/L	73.97 ± 5.25^a^	61.59 ± 2.44^b^
Urine sodium	40–220 mmol/L	95.53 ± 4.48^a^	142.53 ± 4.85^b^

Frequencies of biochemical abnormalities in the study population

The prevalence of biochemical abnormalities was higher among contraceptive users compared with non-users (Table 3). Users showed elevated frequencies of hypercholesterolemia, abnormal HDL-C levels, elevated LDL-C, hypertriglyceridemia (p < 0.001), and elevated CRP (p < 0.009). Electrolyte disturbances were also more prevalent in users, including hypercalcemia, hyperchloremia, hyperkalemia, and hypernatremia in serum, as well as urinary abnormalities such as hypercalciuria, hyperkaluria, and hypochloriuria (p < 0.05).

**Table 3:** Frequencies of biochemical abnormalities according to the contraceptive status *Fisher’s exact test was used due to zero cell count. The Pearson’s chi-square test was used to assess differences in the frequency of biochemical abnormalities across the two groups, with a p-value below 0.05 considered statistically significant. n: number of subjects per group; HDL: high-density lipoprotein; LDL: low-density lipoprotein

Parameters	Reference values	Variations	Users n = 270 n (%)	Non-users n = 240 n (%)	Chi-square	p-value
C-reactive protein	< 6 mg/dL	Hyper	76(28.10)	44 (18.30)	6.80	0.009
		Normal	194 (71.90)	196 (81.70)		
Total cholesterol	< 200 mg/dL	Hyper	145(53.70)	60 (25.00)	43.54	< 0.001
		Normal	125 (46.30)	180 (75.00)		
HDL cholesterol	45–65 mg/dL	Hyper	73 (27.00)	54 (22.00)	72.10	< 0.001
		Hypo	123(45.6)	19 (7.9)		
		Normal	74 (27.4)	167 (69.6)		
LDL cholesterol	< 150 mg/dL	Hyper	104 (38.50)	50 (20.80)	18.85	< 0.001
		Normal	166 (61.50)	190 (79.20)		
Triglycerides	35–135 mg/dL	Hyper	111(41.10)	28(11.70)	57.04	< 0.001
		Hypo	21(7.80)	38 (15.80)		
		Normal	138(51.10)	174(72.50)		
Serum calcium	8.50–10.8 mg/dL	Hyper	126 (46.70)	39(16.30)	124.60	< 0.001
		Hypo	103 (38.10)	29(12.10)		
		Normal	41(15.20)	172 (71.70)		
Serum chloride	98–110 mmol/L	Hyper	159(58.90)	41(17.10)	136.20	< 0.001
		Hypo	93 (34.40)	48 (20.00)		
		Normal	18(6.70)	151(62.90)		
Serum potassium	3.60–5.50 mmol/L	Hyper	124 (45.90)	24 (10.00)	88.70	< 0.001
		Hypo	79 (29.30)	55 (22.90)		
		Normal	67 (24.80)	161(67.10)		
Serum sodium	136–146 mmol/L	Hyper	78 (29.00)	23 (9.60)	90.40	< 0.001
		Hypo	129 (48.00)	49 (20.40)		
		Normal	62 (23.00)	168(70.00)		
Urine calcium	6.69–20.04 mg/dL	Hyper	44 (16.30)	0 (0.00)	–	< 0.001*
		Hypo	84 (31.10)	112 (46.70)		
		Normal	142 (52.60)	128 (53.30)		
Urine chloride	170–250 mmol/L	Hyper	13(4.80)	8(3.30)	10.71	0.005
		Hypo	195 (72.20)	202 (84.20)		
		Normal	62(23.00)	30 (12.50)		
Urine potassium	25–125 mmol/L	Hyper	77 (28.50)	18(7.50)	91.30	< 0.001
		Hypo	124 (45.90)	50 (20.80)		
		Normal	69 (25.60)	172 (71.70)		
Urine sodium	40–220 mmol/L	Hyper	21(7.80)	35(14.60)	34.30	< 0.001
		Hypo	71 (26.30)	6(2.5)		
		Normal	178 (65.90)	199 (82.9)		

Frequencies of biochemical abnormalities according to the type of contraceptive

Analysis by contraceptive type (Table 4) revealed that implants were associated with the highest prevalence of hyperkalemia, hyperchloremia, hypercalcemia, and hypercholesterolemia. Pills and injections also showed significant disturbances, particularly in CRP and lipid profiles (all p < 0.001), indicating that different hormonal formulations have varying metabolic and electrolyte effects.

**Table 4:** Frequencies of biochemical abnormalities according to the type of contraceptive * Fisher’s exact test was used due to small expected cell counts (< 5). The Pearson’s chi-square test was used to assess differences in the frequency of biochemical abnormalities across the three groups, with a p-value below 0.05 considered statistically significant. n: number of subjects per group; HDL: high-density lipoprotein; LDL: low-density lipoprotein

Parameters	Reference values	Variations	Implant n = 198 n (%)	Pill n = 33 n (%)	Injection n = 39 n (%)	Chi-square	p-value
C-reactive protein	< 6 mg/dL	Hyper	64(32.32)	3 (9.10)	9 (23.08)	17.21	< 0.001
		Normal	134 (67.67)	30 (90.90)	30 (76.92)		
Total cholesterol	< 200 mg/dL	Hyper	108(54.54)	23 (69.70)	14 (35.90)	55.68	< 0.001
		Normal	90 (45.45)	10 (30.30)	25 (64.10)		
HDL cholesterol	45–65 mg/dL	Hyper	50 (25.25)	13 (39.39)	13 (33.33)	88.40	< 0.001
		Hypo	91(45.95)	15 (45.45)	19 (48.71)		
		Normal	57 (28.78)	5 (15.15)	7 (17.94)		
LDL cholesterol	< 150 mg/dL	Hyper	78 (39.40)	17 (51.52)	8 (20.52)	26.86	< 0.001
		Normal	120 (60.60)	16 (48.48)	31 (79.48)		
Triglycerides	35–135 mg/dL	Hyper	84(42.43)	11(17.53)	16 (41.03)	56.90	< 0.001
		Hypo	14(7.07)	4 (33.33)	3 (7.69)		
		Normal	100(50.50)	18(54.54)	20 (51.28)		
Serum calcium	8.50–10.8 mg/dL	Hyper	92 (46.46)	18(54.54)	16 (41.02)	126.80	< 0.001
		Hypo	76 (38.38)	11(33.33)	17 (43.58)		
		Normal	30(15.15)	4 (12.12)	6 (15.38)		
Serum chloride	98–110 mmol/L	Hyper	117(59.09)	20 (60.60)	20 (51.28)	138.60	< 0.001
		Hypo	68 (34.34)	10 (30.30)	17 (43.58)		
		Normal	13(6.56)	3(9.09)	2 (5.12)		
Serum potassium	3.60–5.50 mmol/L	Hyper	92 (46.46)	16 (48.48)	17 (43.58)	92.10	< 0.001
		Hypo	64 (32.32)	6 (18.18)	9 (23.07)		
		Normal	42 (21.21)	11(33.33)	13 (33.33)		
Serum sodium	136–146 mmol/L	Hyper	61 (30.80)	8 (24.24)	11 (28.20)	96.30	< 0.001
		Hypo	91 (45.95)	19 (57.57)	19 (48.71)		
		Normal	45 (22.72)	6(18.18)	9 (23.07)		
Urine calcium	6.69–20.04 mg/dL	Hyper	187 (94.44)	31 (93.93)	39 (100)	–	< 0.001*
		Normal	11 (5.55)	2 (6.06)	0 (0)		
Urine chloride	170–250 mmol/L	Hyper	137 (69.19)	28 (84.84)	30 (76.92)	17.61	< 0.001
		Normal	61(30.08)	5 (15.15)	9 (23.07)		
Urine potassium	25–125 mmol/L	Hyper	80 (40.40)	15(45.45)	13 (33.33)	109.8	< 0.001
		Hypo	96 (48.48)	10 (30.30)	18 (46.15)		
		Normal	22 (11.11)	8 (24.24)	8 (20.51)		
Urine sodium	40–220 mmol/L	Hyper	4(2.02)	0(0)	1 (2.56)	–	< 0.001*
		Hypo	103 (52.02)	15(45.45)	23 (58.97)		
		Normal	91 (45.95)	18 (54.54)	15 (38.46)		

Frequencies of biochemical abnormalities according to the duration of use of HCs

Most biochemical abnormalities are significantly associated with HC use duration (Table 5). Lipid abnormalities are frequent, with high total cholesterol, elevated LDL, low HDL, and increased triglycerides, indicating an unfavorable cardiovascular profile. Serum and urinary electrolytes also show widespread imbalances, with both hyper- and hypo-values observed, particularly for calcium, sodium, potassium, and chloride, suggesting disrupted metabolic and renal regulations. However, there is no consistent trend with increasing duration, indicating that HC use itself, rather than duration, is the main factor associated with these biochemical disturbances.

**Table 5:** Frequencies of biochemical abnormalities according to the duration of use of hormonal contraceptives * Fisher’s exact test was used due to small expected cell counts (< 5). The Pearson’s chi-square test was used to assess differences in the frequency of biochemical abnormalities across the four groups, with a p-value below 0.05 considered statistically significant. n: number of subjects per group; HDL: high-density lipoprotein; LDL: low-density lipoprotein

Parameters	Reference values	Variations	3–12 months (n = 165) n (%)	12–24 months (n = 44) n (%)	24–48 months (n = 36) n (%)	≥ 48 months (n = 25) n (%)	Chi-square	p-value
C-reactive protein	< 6 mg/dL	Hyper	48(29.09)	13 (29.54)	10 (27.77)	5 (20)	8.02	0.045
		Normal	117 (70.90)	31 (70.45)	26 (72.22)	20 (80)		
Total cholesterol	< 200 mg/dL	Hyper	87(52.72)	28 (63.63)	16 (44.44)	14 (56)	24.90	< 0.001
		Normal	78 (47.27)	16 (36.36)	20 (55.55)	11 (44)		
HDL cholesterol	45–65 mg/dL	Hyper	50 (30.30)	11 (25)	9 (25)	6 (24)	9.88	0.02
		Hypo	79(47.87)	21 (47.72)	13 (36.11)	12 (48)		
		Normal	36 (21.81)	12 (27.27)	14 (38.88)	7 (28)		
LDL cholesterol	< 150 mg/dL	Hyper	64 (38.78)	18 (40.90)	11 (30.55)	10 (40)	2.61	0.45
		Normal	101 (61.21)	26 (59.09)	25 (69.44)	15 (60)		
Triglycerides	35–135 mg/dL	Hyper	74(44.84)	11(25)	17 (42.22)	9 (36)	9.10	0.028
		Hypo	9(5.45)	7 (15.90)	2 (5.55)	3 (12)		
		Normal	82 (49.69)	26(59.09)	17 (47.22)	13 (52)		
Serum calcium	8.50–10.8 mg/dL	Hyper	79 (47.88)	25(56.81)	11 (30.55)	11 (44)	42.80	< 0.001
		Hypo	64 (38.78)	13(29.54)	18 (50)	9 (36)		
		Normal	22(13.33)	6 (13.63)	7 (19.44)	5 (20)		
Serum chloride	98–110 mmol/L	Hyper	93(56.36)	30 (68.18)	18 (50)	16 (64)	49.10	< 0.001
		Hypo	63 (38.18)	11 (25)	14 (38.88)	7 (28)		
		Normal	9(5.45)	3(6.81)	4 (11.11)	2 (8)		
Serum potassium	3.60–5.50 mmol/L	Hyper	83 (50.30)	21 (47.72)	10 (27.77)	11 (44)	33.70	< 0.001
		Hypo	50 (30.30)	8 (18.18)	15 (41.66)	6 (24)		
		Normal	32 (19.39)	15(34.09)	11 (30.55)	8 (32)		
Serum sodium	136–146 mmol/L	Hyper	55 (33.33)	12 (27.27)	7 (19.44)	7 (28)	37.20	< 0.001
		Hypo	77 (46.66)	22 (50)	15 (41.66)	15 (60)		
		Normal	33 (20)	10(22.72)	14 (38.88)	3 (12)		
Urine calcium	6.69–20.04 mg/dL	Hyper	155 (93.93)	42 (95.45)	36 (100)	24 (96)	–	0.07*
		Normal	10 (6.06)	2 (4.54)	0 (0)	1 (4)		
Urine chloride	170–250 mmol/L	Hyper	6 (3.63)	1 (2.27)	4 (11.11)	1 (4)	–	0.31*
		Hypo	126 (76.36)	30 (68.18)	24 (66.66)	15 (60)		
		Normal	33(20)	13 (29.54)	8 (22.22)	9 (36)		
Urine potassium	25–125 mmol/L	Hyper	74 (44.84)	19(43.18)	7 (19.44)	8 (32)	55.60	< 0.001
		Hypo	70 (42.42)	19 (43.18)	23 (63.88)	12 (48)		
		Normal	21 (12.72)	6 (13.63)	6 (16.66)	5 (20)		
Urine sodium	40–220 mmol/L	Hyper	3(1.81)	1(2.27)	1 (2.77)	0 (0)	–	0.38*
		Hypo	88 (53.33)	20(45.45)	18 (50)	15 (60)		
		Normal	74 (44.84)	23 (52.27)	17 (47.22)	10 (40)		

Association between biochemical abnormalities and the status of users or non-users of HCs of the study participants

HC use is significantly associated with several biochemical abnormalities (Table 6). HC users have higher levels of CRP, total cholesterol, and LDL, indicating increased inflammation and dyslipidemia, while low HDL is also more frequent, suggesting a less favorable cardiovascular profile. Electrolyte imbalances are pronounced among users, with significant associations for calcium, chloride, potassium, and sodium disturbances, both high and low. These findings suggest altered metabolic and renal regulation. Urinary changes are less consistent and limited to calcium abnormalities, though elevated urinary potassium is associated with HC use, while urinary sodium and chloride show an inverse relationship.

**Table 6:** Biochemical abnormalities associated with hormonal contraceptive use A multivariate logistic regression analysis was conducted to examine the associations between hormonal contraceptive use and biochemical abnormalities; a p-value less than 0.05 was considered statistically significant. An odds ratio (OR) of 1 indicates no association; an OR greater than 1 suggests the predictor is a risk factor, and an OR less than 1 indicates the predictor is protective. n: number of subjects per group; HDL: high-density lipoprotein; LDL: low-density lipoprotein

Parameters	Reference values	Variations	Users n = 270	
			p-value	Odd ratio (confidence interval)
C-reactive protein	< 6 mg/dL	Hyper	0.001	3.18 (2.07–10.45)
		Normal	-	Reference
Total cholesterol	< 200 mg/dL	Hyper	0.001	4.57 (1.59–13.13)
		Normal	-	Reference
HDL cholesterol	45–65 mg/dL	Hyper	0.42	0.70 (0.30–1.65)
		Hypo	0.01	3.17 (1.21–8.32)
		Normal	-	Reference
LDL cholesterol	< 150 mg/dL	Hyper	0.001	2.80 (1.27–7.60)
		Normal	-	Reference
Triglycerides	35–135 mg/dL	Hyper	0.19	1.51 (0.84–3.39)
		Hypo	0.001	0.10 (0.03–0.37)
		Normal	-	Reference
Serum calcium	8.50–10.8 mg/dL	Hyper	0.01	3.68 (1.26–10.77)
		Hypo	0.001	23.62 (9.10–46.29)
		Normal	-	Reference
Serum chloride	98–110 mmol/L	Hyper	0.001	11.23 (4.32–23.17)
		Hypo	0.001	4.79 (1.88–8.23)
		Normal	-	Reference
Serum potassium	3.60–5.50 mmol/L	Hyper	0.001	6.86 (2.60–15.13)
		Hypo	0.01	2.57 (1.18–6.59)
		Normal	-	Reference
Serum sodium	136–146 mmol/L	Hyper	0.001	7.20 (2.93–17.73)
		Hypo	0.05	2.30 (0.93–5.33)
		Normal	-	Reference
Urine calcium	6.69–20.04 mg/dL	Hyper	0.001	5.12 (1.80–12.56)
		Normal	-	Reference
Urine chloride	170–250 mmol/L	Hypo	0.29	0.64 (0.28–1.46)
		Normal	-	Reference
Urine potassium	25–125 mmol/L	Hyper	0.001	5.42 (2.16–13.61)
		Hypo	0.10	2.21 (0.83–5.88)
		Normal	-	Reference
Urine sodium	40–220 mmol/L	Hyper	0.38	0.4 (0.23–1.26)
		Hypo	0.28	1.42 (0.74–2.71)
		Normal	-	Reference

Association between biochemical abnormalities and the type of HC

Biochemical abnormalities vary by contraceptive type, with implants showing the strongest associations overall (Table 7). CRP is significantly associated with implants. Elevated total cholesterol is linked to implants and pills, while LDL changes are significant across both implants and pills. Low HDL is more evident with all methods. Triglyceride elevations are mainly associated with implants and pills. Electrolyte disturbances are prominent, especially across all methods. Urinary changes are limited to hypercalciuria and high potassium, though implants are associated with reduced potassium and high sodium levels. Overall, implants have the greatest effect on biochemical parameters, followed by pills, while injectables show fewer effects.

**Table 7:** Association between biochemical abnormalities and the type of hormonal contraceptives A multivariate logistic regression analysis was conducted to examine the associations between types of hormonal contraceptives and biochemical abnormalities; a p-value less than 0.05 was considered statistically significant. An odds ratio (OR) of 1 indicates no association; an OR greater than 1 suggests the predictor is a risk factor, and an OR less than 1 indicates the predictor is protective. n: number of subjects per group; HDL: high-density lipoprotein; LDL: low-density lipoprotein

Parameters	Reference values	Variations	Implant n = 198		Pill n = 33		Injection n = 39	
			p-value	Odds ratio (confidence interval)	p-value	Odds ratio (confidence interval)	p-value	Odds ratio (confidence interval)
C-reactive protein	< 6 mg/dL	Hyper	0.001	3.63 (2.11–6.24)	0.97	0.98 (0.28–3.35)	0.19	1.72 (0.75–3.93)
		Normal		Reference	-	Reference	-	Reference
Total cholesterol	< 200 mg/dL	Hyper	0.004	3.33 (1.48–7.51)	0.001	7.38 (3.28–16.58)	0.10	1.81 (0.89–3.69)
		Normal		Reference	-	Reference	-	Reference
HDL cholesterol	45–65 mg/dL	Hyper	0.01	1.92 (1.14–3.22)	0.25	1.65 (0.70–3.89)	0.40	1.41 (0.63–3.14)
		Hypo	0.001	8.25 (4.80–14.71)	0.001	8.10 (3.41–19.22)	0.001	8.84 (3.95–19.78)
		Normal		Reference	-	Reference	-	Reference
LDL cholesterol	< 150 mg/dL	Hyper	0.001	2.49 (1.61–3.86)	0.001	4.00 (1.82–8.77)	0.52	1.31 (0.57–2.98)
		Normal		Reference	-	Reference	-	Reference
Triglycerides	35–135 mg/dL	Hyper	0.001	5.57 (3.43–9.04)	0.002	3.78 (1.60–8.90)	0.001	5.27 (2.37–11.72)
		Hypo	0.35	0.72 (0.36–1.44)	0.38	0.58 (0.17–1.98)	0.72	0.81 (0.25–2.59)
		Normal		Reference	-	Reference	-	Reference
Serum calcium	8.50–10.8 mg/dL	Hyper	0.001	4.47 (2.80–7.13)	0.001	5.47 (2.43–12.30)	0.001	4.02 (1.88–8.59)
		Hypo	0.001	4.62 (2.75–7.76)	0.002	3.88 (1.67–9.01)	0.001	4.11 (1.84–9.17)
		Normal		Reference	-	Reference	-	Reference
Serum chloride	98–110 mmol/L	Hyper	0.001	7.23 (4.57–11.44)	0.001	10.20 (4.16–25.01)	0.001	4.89 (2.23–10.73)
		Hypo	0.001	3.44 (2.10–5.64)	0.003	3.80 (1.56–9.24)	0.01	2.95 (1.27–6.89)
		Normal		Reference	-	Reference	-	Reference
Serum potassium	3.60–5.50 mmol/L	Hyper	0.001	8.18 (4.82–13.87)	0.001	8.85 (3.63–21.55)	0.001	7.38 (3.26–16.68)
		Hypo	0.001	3.40 (2.06–5.61)	0.01	2.95 (1.28–6.78)	0.004	3.12 (1.41–6.88)
		Normal		Reference	-	Reference	-	Reference
Serum sodium	136–146 mmol/L	Hyper	0.01	4.13 (2.40–7.10)	0.001	4.75 (1.97–11.45)	0.01	3.22 (1.32–7.86)
		Hypo	0.01	4.50 (2.75–7.36)	0.001	4.11 (1.76–9.58)	0.002	3.76 (1.66–8.49)
		Normal		Reference	-	Reference	-	Reference
Urine calcium	6.69–20.04 mg/dL	Hyper	0.001	12.85 (4.95–36.71)	0.001	15.92 (4.88–51.94)	0.001	18.43 (5.21–65.18)
		Normal		Reference	-	Reference	-	Reference
Urine chloride	170–250 mmol/L	Hypo	0.18	1.28 (0.34–4.86)	0.70	1.28 (0.34–4.86)	0.55	0.71 (0.23–2.18)
		Normal		Reference	-	Reference	-	Reference
Urine potassium	25–125 mmol/L	Hyper	0.01	5.21 (3.11–8.72)	0.001	4.73 (2.05–10.91)	0.001	3.88 (1.75–8.59)
		Hypo	0.027	3.17 (1.13–8.84)	0.61	0.65 (0.12–3.37)	0.84	1.16 (0.27–5.00)
		Normal		Reference	-	Reference	-	Reference
Urine sodium	40–220 mmol/L	Hyper	0.01	0.21 (0.06–0.72)	0.39	0.38 (0.04–3.52)	0.09	0.30 (0.07–1.20)
		Hypo	0.34	1.38 (0.70–2.70)	0.45	0.67 (0.24–1.88)	0.15	1.98 (0.78–5.04)
		Normal		Reference	-	Reference	-	Reference

Association between biochemical abnormalities and the duration of use of HCs

Both short- and long-term use of HC is associated with biochemical abnormalities (Table 8). CRP is elevated with implants, indicating persistent inflammation. LDL is higher in early and prolonged use, while high total cholesterol, elevated triglycerides, and low HDL are seen across all durations. Electrolyte disturbances are notable at all durations, especially hypocalcemia, and imbalances in chloride, potassium, and sodium, with some effects persisting beyond 48 months. Urinary changes are mainly seen in early users, particularly for calcium, potassium, and sodium. Overall, HC use leads to sustained inflammation, lipid alterations, and electrolyte disturbances, emphasizing the need for regular monitoring throughout use.

**Table 8:** Association of biochemical abnormalities according to the duration of hormonal contraceptive use A multivariate logistic regression analysis was conducted to examine the associations between the duration of hormonal contraceptive use and biochemical abnormalities; a p-value less than 0.05 was considered statistically significant; an odds ratio (OR) of 1 indicates no association; an OR greater than 1 suggests the predictor is a risk factor, and an OR less than 1 indicates the predictor is protective. n: number of subjects per group; HDL: high-density lipoprotein; LDL: low-density lipoprotein

Parameters	Reference values	Variations	3–12 months (n = 165)		12–24 months (n = 44)		24–48 months (n = 36)		≥ 48 months (n = 25)	
			p-value	Odds ratio (confidence interval)	p-value	Odds ratio (confidence interval)	p-value	Odds ratio (confidence interval)	p-value	Odds ratio (confidence interval)
C-reactive protein	< 6 mg/dL	Hyper	0.01	1.96 (1.17–3.28)	0.06	2.01 (0.97–4.16)	0.12	1.85 (0.84–4.06)	0.55	1.37 (0.48–3.90)
		Normal	-	Reference	-	Reference	-	Reference	-	Reference
Total cholesterol	< 200 mg/dL	Hyper	0.01	3.64 (2.40–5.52)	0.001	5.63 (2.91–10.89)	0.009	2.58 (1.26–5.28)	0.001	4.06 (1.75–9.39)
		Normal	-	Reference	-	Reference	-	Reference	-	Reference
HDL cholesterol	45–65 mg/dL	Hyper	0.04	1.70 (1.02–2.84)	0.24	1.60 (0.72–3.53)	0.18	1.80 (0.75–4.31)	0.38	1.50 (0.60–3.75)
		Hypo	0.001	8.02 (4.54–14.17)	0.001	6.72 (3.00–15.05)	0.001	8.25 (3.47–19.60)	0.001	10.10 (3.98–25.61)
		Normal	-	Reference	-	Reference	-	Reference	-	Reference
LDL cholesterol	< 150 mg/dL	Hyper	0.01	2.38 (1.51–3.76)	0.005	2.66 (1.33–5.33)	0.19	1.67 (0.78–3.58)	0.03	2.53 (1.05–6.09)
		Normal	-	Reference	-	Reference	-	Reference	-	Reference
Triglycerides	35–135 mg/dL	Hyper	0.001	5.93 (3.60–9.76)	0.01	2.52 (1.20–5.27)	0.001	6.04 (2.84–12.85)	0.002	4.25 (1.73–10.42)
		Hypo	0.001	0.06 (0.016–0.26)	0.012	0.14 (0.03–0.65)	0.061	1.36 (0.36–4.99)	0.126	0.23 (0.03–1.51)
		Normal	-	Reference	-	Reference	-	Reference	-	Reference
Serum calcium	8.50–10.8 mg/dL	Hyper	0.040	3.46 (1.06–11.30)	0.009	7.99 (1.67–38.09)	0.146	3.25 (0.66–15.93)	0.272	2.69 (0.46–15.72)
		Hypo	0.001	24.50 (8.63–69.54)	0.001	24.29 (6.20–95.07)	0.001	21.25 (5.95–75.80)	0.001	17.75 (3.94–79.90)
		Normal	-	Reference	-	Reference	-	Reference	-	Reference
Serum chloride	98–110 mmol/L	Hyper	0.001	10.47 (3.35–32.78)	0.001	15.54 (2.92–82.65)	0.004	9.03 (2.01–40.48)	0.015	11.57 (1.59–83.80)
		Hypo	0.002	5.94 (1.96–17.93)	0.123	3.75 (0.69–20.24)	0.116	3.36 (0.74–15.27)	0.300	2.88 (0.38–21.41)
		Normal	-	Reference	-	Reference	-	Reference	-	Reference
Serum potassium	3.60–5.50 mmol/L	Hyper	0.001	9.68 (3.35–27.94)	0.025	4.43 (1.20–16.33)	0.730	3.71 (0.88–15.53)	0.024	5.93 (1.26–27.85)
		Hypo	0.004	3.54 (1.50–8.34)	0.846	1.12 (0.34–3.64)	0.910	2.51 (0.88–7.36)	0.484	1.62 (0.41–6.26)
		Normal	-	Reference	-	Reference	-	Reference	-	Reference
Serum sodium	136–146 mmol/L	Hyper	0.001	9.86 (3.71–26.17)	0.002	7.00 (2.00–24.41)	0.374	1.83 (0.48–6.96)	0.001	14.97 (2.92–76.78)
		Hypo	0.072	2.30 (0.92–5.72)	0.165	2.20 (0.82–6.71)	0.659	1.29 (0.41–3.99)	0.019	6.31 (1.35–29.33)
		Normal	-	Reference	-	Reference	-	Reference	-	Reference
Urine calcium	6.69–20.04 mg/dL	Hyper	0.001	10.42 (3.95–27.46)	0.003	6.88 (2.01–23.54)	0.010	4.95 (1.45–16.90)	0.001	8.12 (2.23–29.58)
		Normal	-	Reference	-	Reference	-	Reference	-	Reference
Urine chloride	170–250 mmol/L	Hypo	0.557	0.76 (0.32–1.84)	0.42	0.65 (0.22–1.85)	0.308	0.57 (0.19–1.67)	0.074	0.34 (0.10–1.10)
		Normal	-	Reference	-	Reference	-	Reference	-	Reference
Urine potassium	25–125 mmol/L	Hyper	0.001	7.784 (2.80–21.64)	0.042	4.36 (1.05–18.07)	0.123	3.21 (0.72–14.22)	0.332	2.24 (0.43–11.51)
		Hypo	0.144	2.24 (0.75–6.65)	0.256	2.39 (0.53–10.79)	0.126	3.08 (0.72–13.02)	0.735	1.34 (0.24–7.57)
		Normal	-	Reference	-	Reference	-	Reference	-	Reference
Urine sodium	40–220 mmol/L	Hyper	0.002	0.03 (0.01–0.28)	0.011	0.02 (0.01–0.43)	0.142	0.25 (0.14–1.84)	0.991	0.70 (0.43–7.58)
		Hypo	0.222	1.54 (0.76–3.10)	0.997	1.08 (0.43–2.62)	0.622	1.25 (0.50–3.12)	0.234	1.88 (0.66–5.34)
		Normal	-	Reference	-	Reference	-	Reference	-	Reference

## Discussion

The present investigation demonstrated that the use of HCs was associated with significant alterations in several biochemical parameters. Compared with non-users, HC users exhibited significantly higher levels of CRP, total cholesterol, LDL cholesterol, triglycerides, and several blood and urinary electrolytes, including calcium, chloride, and potassium (p < 0.001). These findings suggest that HC use may influence metabolic processes and affect organ systems involved in homeostasis, particularly the cardiovascular, hepatic, and renal systems.

The observed differences in lipid profile parameters among HC users may be explained by the hormonal components of contraceptives. Estrogens generally increase HDL cholesterol and decrease LDL cholesterol levels, whereas progestins may exert the opposite effect by lowering HDL and increasing LDL concentrations [10]. Previous studies have also reported that HCs can elevate triglyceride levels, often representing the most pronounced lipid alteration associated with contraceptive use [11]. Such disturbances in lipid metabolism may contribute to the development of cardiovascular conditions such as atherosclerosis, coronary artery disease, and stroke.

In addition to lipid abnormalities, significant changes in electrolyte levels were observed among HC users. Hormonal influences on renal physiology and fluid regulation may account for these alterations. Estrogen can affect renal filtration and electrolyte balance, potentially leading to variations in serum and urinary electrolyte concentrations.

CRP levels were also significantly higher among HC users, indicating increased inflammatory or metabolic activity. CRP is widely recognized as a marker of systemic inflammation and a predictor of cardiovascular risk. However, some studies suggest that the CRP elevation associated with oral contraceptive use may reflect a metabolic response to estrogen rather than a direct inflammatory process [12,13]. Estrogen may stimulate immune-related processes such as antibody synthesis, while progesterone may suppress certain immune responses, including T-lymphocyte activity.

The association between HC use and cardiovascular risk has been widely documented. Combined HCs have been linked to an increased risk of thromboembolic events, including pulmonary embolism, deep vein thrombosis, and superficial venous thrombosis [14]. The magnitude of this risk is influenced by the estrogen dose, with higher doses associated with a greater likelihood of venous thromboembolic disease [4,15]. Additionally, combined contraceptives may cause slight increases in systolic and diastolic blood pressure, with hypertension reported in 0.6-8.5% of users [16].

In the present study, biochemical abnormalities were more frequently observed among implant users than among users of other contraceptive methods. Furthermore, these abnormalities were more common among women who had used HC for less than one year. This may reflect early physiological responses to hormonal exposure, as HCs suppress gonadotropin secretion and modify the hypothalamic-pituitary-gonadal axis, potentially affecting immune and metabolic regulation [17].

Overall, these findings confirm that HCs may alter biochemical homeostasis beyond their contraceptive function. Consistent with previous reports [12,13], HC users showed increased CRP levels and metabolic changes affecting lipid and electrolyte balance. Implant users appeared particularly susceptible to these alterations, which may be related to sustained hormonal exposure and hepatic effects on lipid metabolism, as previously reported [18].

Given that these metabolic changes may occur without overt clinical symptoms, biochemical screening prior to initiating hormonal contraception and periodic monitoring during use may be beneficial. Regular evaluation of lipid profiles, inflammatory markers, and electrolyte levels could help identify early metabolic disturbances and ensure the safe use of HC methods.

This study has several strengths that enhance the robustness of its findings. It addresses an important but under-researched topic in sub-Saharan Africa, with a reasonably adequate sample size that improves statistical power. The comprehensive assessment of multiple biochemical parameters, including lipid profile, CRP, and electrolytes, provides a broad evaluation of potential systemic effects of HC use. In addition, stratification by contraceptive type and duration of use allowed for more detailed analysis of variations in biochemical responses. Overall, these strengths support the relevance of the findings for clinical and public health practice in Cameroon and similar settings.

Limitations

Several limitations should be considered when interpreting the findings of this study. First, although data verification procedures were strengthened and all biochemical values were revalidated against the original laboratory records, the possibility of measurement or data-handling errors cannot be completely excluded. Second, despite adjustments for age, physical activity, and BMI, residual confounding from unmeasured factors, such as dietary habits, socioeconomic status, genetic predisposition, duration and adherence to contraceptive use, and other lifestyle characteristics, may have influenced the observed associations. In addition, the imbalance in subgroup sizes, particularly among implant users, may have affected the precision and comparability of subgroup analyses. Finally, the cross-sectional design limits the ability to establish temporal relationships or infer causality between HC use and biochemical alterations. Therefore, the findings should be interpreted cautiously and confirmed through longitudinal studies with repeated biochemical assessments.

## Conclusions

Overall, this study suggests an association between HC use among women attending the Dschang Regional Annex Hospital and differences in selected biochemical parameters, including lipid profile components, CRP, and electrolyte levels. Some variations appeared more pronounced among implant users. These findings indicate potential metabolic and inflammatory differences related to HC use. The findings nonetheless highlight the importance of considering biochemical monitoring and individual risk assessment within family planning services to support the safe use of HC methods in this population.
